# Demonstration of phage inhibitory action against *Clostridium perfringens* LMG 11264 within a complex chicken cecal microbiota *in vitro*

**DOI:** 10.3389/frabi.2025.1599939

**Published:** 2025-09-18

**Authors:** Maria Wiese, Eline S. Klaassens, Volmar Hatt, Angelique Kreikamp, Mirna L. Baak, Margreet Heerikhuisen, Jos M. B. M. Van Der Vossen

**Affiliations:** ^1^ Microbiology and Systems Biology, Toegepast-Natuurweteschappelijk Onderzoek (TNO), Microbiology and Systems Biology, Leiden, Netherlands; ^2^ Baseclear B.V., Product Development, Leiden, Netherlands

**Keywords:** phage, *Clostridium perfringens*, antibiotic resistance, chicken cecal microbiota, *in vitro*

## Abstract

**Introduction:**

*Clostridium perfringens* strains may cause foodborne illness, and 95% of human infections are linked to the consumption of contaminated meat, including chicken products. In poultry, *C. perfringens* infection may cause necrotic enteritis, and infections are associated with high mortality rates partially due to antibiotic resistance, which hampers efficient treatment. *In-vitro* screening approaches of alternative treatment options, for instance, specific phages, represent a promising strategy for the selection of novel interventions to combat infections.

**Material and methods:**

In this study, we explored the application of a *C. perfringens* strain LMG 11264-specific phage #7 introduced at 10^4^ pfu/mL to inhibit the growth of *C. perfringens* at 10^6^ cfu/mL compared to two antibiotics (amoxicillin at 10 µg/mL and clindamycin at 10 µg/mL) within complex chicken cecal microbiota *in vitro.* Samples for gDNA isolation, qPCR, and metagenome sequencing were taken at the beginning and after 24 and 48 h of incubation.

**Results:**

The *C. perfringens* strain LMG 11264 proliferated within the untreated complex microbiota and reached levels of approximately 10^8^ and 10^9^ genome equivalents per mL after 24 and 48 h of incubation, respectively. The phage intervention with phage #7 inhibited the growth of *C. perfringens* LMG 11264 significantly; the inhibitory effects were similar to those exerted by the antibiotic intervention with amoxicillin and stronger than the inhibitory effects with clindamycin. In the absence of the *C. perfringens* challenge, we found a significant effect of amoxicillin (*p* = 0.040) or clindamycin (*p* = 0.000017) compared to the untreated control after 24 h of incubation, and the phage addition did not affect the alpha diversity expressed as Chao index significantly (*p* = 1). In addition, the endogenous *C. perfringens* in the chicken microbiota appeared insensitive to phage #7. The phage titer of phage #7 only increased in the presence of the inoculated *C. perfringens* strain LMG 11264. In conclusion, the i-screen model can be implemented to test the efficacy and specificity of phage therapy *in vitro.*

## Introduction

*Clostridium perfringens* is a spore-forming bacterium, and its vegetative cells may thrive as commensals or potential pathogens within the chicken intestinal tract. Enteritis caused by pathogenic *C. perfringens* strains can be associated with detrimental effects on poultry production (Immerseel et al., 2004). *Clostridium perfringens* strains may produce various virulence factors, such as enzymes and toxins that inflict lesions and may lead to severe infection symptoms. Toxic strains are classified into five toxin types (A–E), of which toxin type A produces the alpha toxin and is the main cause of the subclinical form of infection and necrotic enteritis in poultry (Songer and Meer, 1996; Petit et al., 1999; Immerseel et al., 2004). The A toxin is often produced at the moment of sporulation and is hence frequently also associated with *C. perfringens-*inflicted foodborne disease in humans (McClane et al., 2012).

The use of antibiotic growth factors was an integral part of the management measures for increased productivity and protection against necrotic enteritis in the past. However, antibiotic growth factors were banned (Casewell et al., 2003), and consequently, alternative management strategies are being explored.

The chicken gastrointestinal tract harbors a diverse microbiota that plays an essential role in gut and overall health; it aids in the digestion of feed and plays a pivotal role in colonization resistance, detoxification, and modulation of immune system development (Mead, 1997; Apajalahti et al., 2004; Wei et al., 2013). Therefore, advanced sustainable antimicrobial treatments that spare the beneficial function of the endogenous gut microbiome are desirable. Nevertheless, such microbiome-friendly treatments are still widely missing. For the containment of *C. perfringens* next to more elaborate dietary and management practices, alternative solutions such as biotics, competitive exclusion products, enzymes, organic acids, plant extracts, bacteriophages, antibodies, and vaccination are being explored (Dahiya et al., 2006; M’Sadeq et al., 2015).

Various *C. perfringens-*specific bacteriophages have been described and were, e.g., reviewed by Seal et al (Seal et al., 2012; Venhorst et al., 2022). A recent review also reported on the advances in bacteriophages as promising alternatives to control zoonotic pathogens in animals and food (Gambino and Brøndsted, 2021). Bacteriophages or bacteriophage-derived enzymes displaying activity against *C. perfringens* strains have been tested for the control of *C. perfringens* in poultry (Venhorst et al., 2022), e.g., by Zimmer et al., who described the benefits of the application specificity of a murein hydrolase, which lysed all tested *C. perfringens* strains in their study, sparing other bacterial genera and clostridial species (Zimmer et al., 2002). Others have also reported promising results when testing phages *in vivo*, e.g., Miller et al. studied a cocktail of bacteriophages (INT-401) for the potential control of necrotic enteritis caused by *C. perfringens*. Their phage treatment reduced pathogen-inflicted mortality and improved feed conversion ratios and weight gain in the *C. perfringens*-challenged chickens compared to the phage-untreated control birds (Miller et al., 2010). Nevertheless, the use of bacteriophage for *in-vivo* control of *C. perfringens* in poultry still needs to be further investigated (Immerseel et al., 2004; Gigante and Atterbury, 2019). Some of the still poorly understood mechanistic aspects of phage treatments, summed up as the kinetics of self‐replicating pharmaceuticals, have been discussed by Payne et al (Payne and Jansen, 2000). Further studies of the nature of phage and bacterial host dynamics are essential for advances in bacteriophage applications.

Apart from *in-vivo* studies, *in-vitro* studies may provide valuable mechanistic insights into the kinetics of phage replication. A plethora of *in-vitro* models with varying levels of complexity and throughput for the simulation of the chicken gastrointestinal tract have been developed and applied (Miller et al., 2010; Card et al., 2017; Priyodip and Balaji, 2019; Feye et al., 2020; Asare et al., 2021; de Carvalho et al., 2021; Oost et al., 2021; Forssten et al., 2023; Olson et al., 2024). These range from simple batch fermentation setups (Feye et al., 2020) to continuous-flow *in-vitro* fermentation models (Card et al., 2017; Gong et al., 2019; Asare et al., 2021; Oost et al., 2021). *In vitro*, experimental approaches allow the study of pathogen challenge dynamics within complex gut microbial communities (Freeman et al., 2003; Wiese et al., 2022; Wiese et al., 2024). Such models may be used to test novel antimicrobials next to commonly used antibiotics for optimized treatment options and improved gut health. *In-vitro* gut models can be applied to study phage efficacy and specificity within microbiomes of humans (Pinto et al., 2022; Laforêt et al., 2023) and/or animal origin (Chicken PolyFermS) (Asare et al., 2021). The value of gastrointestinal *in-vitro* models for the poultry industry and feed formulations was recently emphasized in a review by de Carvalho et al (de Carvalho et al., 2021).

In this study, we used a 96-well plate-based experimental approach referred to as the i-screen to test the application of a *C. perfringens* strain LMG 11264-specific phage, next to the antibiotics amoxicillin and clindamycin within a complex chicken cecal microbiota *in vitro*. We analyzed the treatment effects on the specificity and efficacy of growth inhibition of the target *C. perfringens* strain, as well as accompanying effects on the overall cecal microbiota.

## Material and methods

### Isolation of phages and specificity testing against *Clostridium perfringens* strains

*Clostridium perfringens* phages were isolated based on the method described by Pedersen et al. (2020) from 10 chicken dissected intestines obtained from a slaughterhouse in Zevenhuizen, the Netherlands, and 12 surface water samples collected from a small pond at Borneoplein, Amersfoort, the Netherlands. In detail, 10 g of intestinal material was homogenized in 90 mL of 0.9% sodium chloride for 2 min. The suspension was centrifuged for 10 min at 10,000×*g*, and subsequently, the supernatant was filtered through a Millex-HV 0.45-µM syringe filter (Sigma-Aldrich, Germany). The centrifugation and filtration steps were also used for the surface water samples. Volumes of 500 µL of filtrate were anaerobically incubated in a diluted suspension of 100 times diluted *C. perfringens* in 10 mL of brain heart infusion (BHI) medium (Oxoid, Thermo Fisher Scientific, Netherlands) for 2 h at 37°C. After centrifugation of the culture for 15 min at 5,000 rpm and filtration through the Millex-HV 0.45-µM filter, 100 µL of the filtrate was mixed with 100 µL overnight culture of *C. perfringens* LMG 11264 (The strain was obtained from the Belgium Culture Collection BCCM/LMG, Gent, Belgium). The mixture was grown in 4 mL of BHI supplemented with 0.4% agarose, 10 mM of MgCl_2_, and CaCl_2_ at approximately 48°C and poured on top of a BHI agar plate. After solidification of the agarose, the plates were incubated overnight at 37°C under microaerophilic conditions using CampyGen (CN0025, Oxoid; Thermo Fisher Scientific, Netherlands), followed by counting plaques. For pure phage preparations, single plaques were picked and processed twice through the above-described infection, centrifugation, and filtration procedure. Phage specificity was additionally tested on a panel of *C. perfringens* strains: *C. perfringens* ATTC 13124, *C. perfringens* DSM 11781, *C. perfringens* LMG 12225, *C. perfringens* LMG 12224, and *C. perfringens* LMG 10468.

### Phage for gDNA extraction

Genomic DNA was extracted from the phage samples using 500 µL of sample material, which was centrifuged for 1 min at 14,000 rpm, and the supernatant was treated with DNase I (Thermo Scientific) for 30 min at 37°C without shaking. A total of 400 µL of DNA/RNA shield (Zymo Research Corporation; Los Angeles, CA, USA) was added, and the samples were transferred to ZR BashingBead Lysis Tubes (0.1 and 0.5 mm). Samples were vortexed on the Vortex-Genie (Scientific Industries) for 15 min at maximum speed. The samples were further extracted using the ZymoBIOMICS DNA miniprep kit (Zymo) according to the manufacturer’s protocol.

### Methods for phage genome analysis

Paired-end sequencing reads were generated using the Illumina NovaSeq 6000 platform. FASTQ files were produced using bcl2fastq version 2.20 (Illumina). An initial quality assessment was conducted based on reads passing Illumina Chastity filtering. Reads containing PhiX control signals were subsequently removed using an in-house filtering protocol. Additionally, reads with partial or full adapter sequences were clipped. A second quality assessment was performed on the remaining reads using FASTQC version 0.11.8. To further improve read quality, BayesHammer (Nikolenko et al., 2013) error correction was applied. The Illumina reads were then aligned to the *Clostridium perfringens* reference strain ATCC^®^ 13124 using Bowtie 2 v2.3.4.5 (Langmead and Salzberg, 2012). It is noteworthy that BCCM/LMG links strain LMG 11264 to ATCC 13124. The resulting alignments were processed using SAMTools 1.9 (Danecek et al., 2021) and BBmap v38.79 (Bushnell, 2014) to remove host bacterial reads. Unaligned reads were subsampled to 6 MB (equivalent to 100× coverage of the phage genome). These reads were assembled into contigs using SPAdes version 3.10 (Bankevich et al., 2012). The order and distance between contigs were estimated from insert size information derived from aligning the paired-end reads to the draft assembly. Contigs were linked and scaffolded using SSPACE version 2.3 (Boetzer et al., 2011). Gaps within scaffolds were partially closed using GapFiller version 1.10 (Boetzer and Pirovano, 2012). Assembly errors and nucleotide discrepancies between Illumina reads and scaffold sequences were corrected using Pilon version 1.21 (Walker et al., 2014). To calculate average nucleotide identity (ANI), the FastANI algorithm (Jain et al., 2018) was applied with a custom database of virus genomes (NCBI Genome Database on 24 January 2023) with selection criteria for complete assemblies. FastANI first divides the query genome into non-overlapping fragments, mapping them to the reference genomes in the database. Alignments, identity estimates, and mappings are then computed for each fragment, and the final ANI is reported. For species-level identification, an ANI threshold of >95% was used, with a fragment length threshold set at 200 bp (Deng et al., 2022).

### Chicken cecal microbiome collection

The inoculum of the i-screen consisted of pooled microbiota material collected from the dissected ceca of poultry birds (Ross 308) that were slaughtered at 6 weeks after hatch. Upon slaughter at Clazing, the Netherlands, the intestine packages were dissected from the birds and immediately individually deposited into a jar made anaerobic with AnaeroGen pack (AN0025 or AN0035, Oxoid). Subsequently, the jars were transported to TNO at ambient temperature. Upon arrival, the cecum content of individual birds, approximately 4 to 13 g, was suspended in 20 mL of anaerobic standard ileum effluent medium (SIEM) supplemented with 5 mL of glycerol. The suspension was slurried under anaerobic conditions using a blender in an anaerobic cabinet, after which 1 mL of aliquots of the slurries were frozen at −80°C.

### *In-vitro* experimentation with chicken microbiota in the i-screen

**Figure 1:**
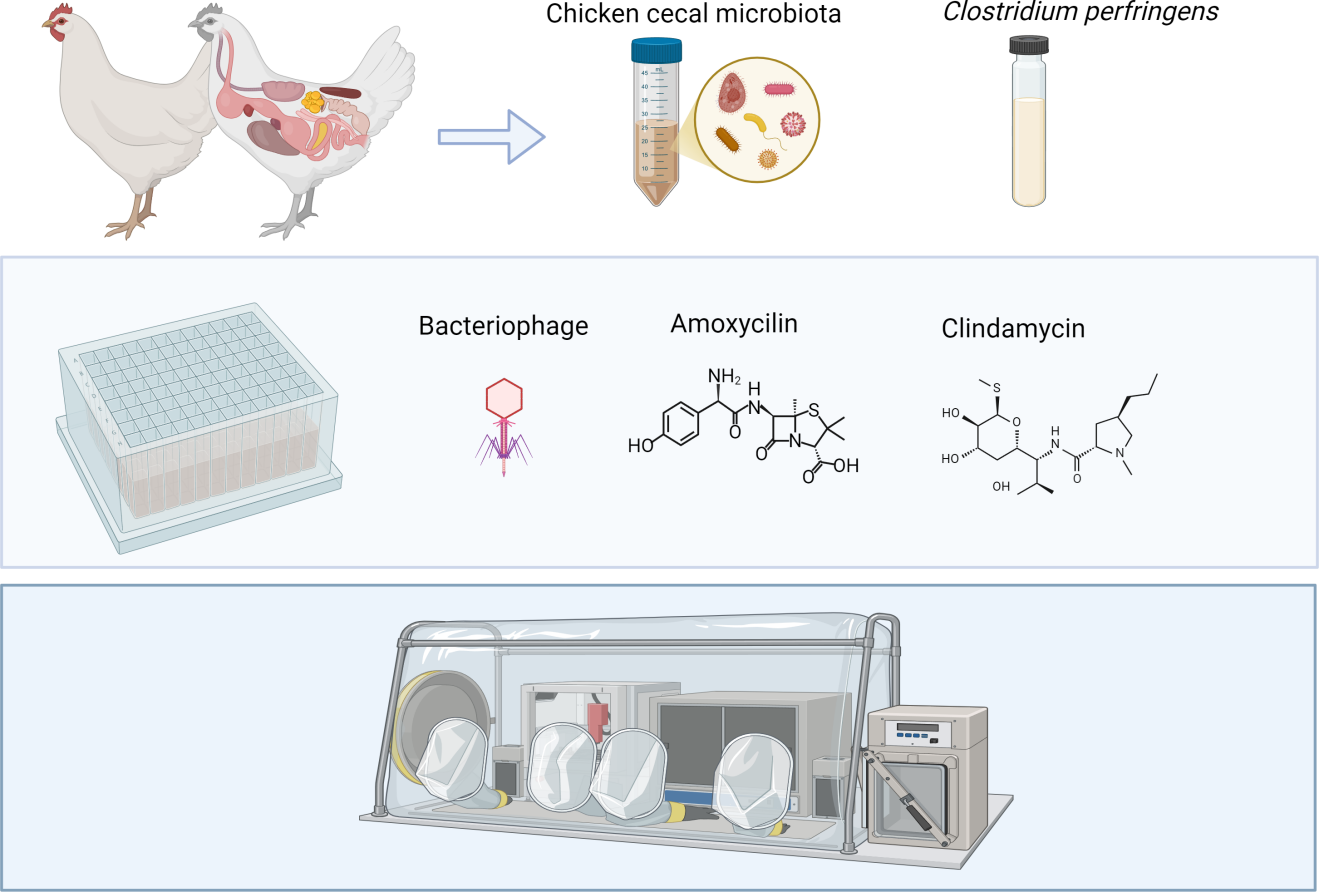
Workflow overview: collection of chicken cecal microbiota and anaerobic culturing of the target pathogen in brain heart infusion medium (*Clostridium perfringens* strain ATCC 13124) and preparation of SIEM media and i-screen *in-vitro* experimental setup based on 96-well plates in anaerobic conditions with and without phage and antibiotics. Anaerobic workstation for the workflow and incubation under microaerophilic conditions (6% O_2_, 75.2% N_2_, 9.4% H_2_, and 9.4% CO_2_), 100 rpm at 41°C. The figure was created with BioRender.com.

In brief, the chicken i-screen experiments representing the conditions of the cecum were performed using a standard SIEM medium (Ladirat et al., 2013) under microaerophilic conditions (6% O_2_, 75.2% N_2_, 9.4% H_2_, and 9.4% CO_2_), 100 rpm at 41°C. The microaerophilic conditions were established using the Anoxomat model AN3 (Advanced Instruments, Norwood, USA). The frozen cecal microbiota of one bird (Clazing no1) with a volume of 200 µL was inoculated in 4.8 mL of SIEM and incubated overnight under the above-described conditions. In addition, an overnight culture of *C. perfringens* LMG 11264 (= ATCC 13124) in BHI (Oxoid) was prepared by culturing under microaerophilic conditions, 100 rpm, and 41°C. At *T* = 0, the i-screen was inoculated with all of the experimental conditions shown in **Table 1** in triplicate with a 50 times diluted cecal microbiota overnight culture in SIEM (**Figure 1**).

**Table 1:** Experimental conditions in the i-screen.

Number	Condition
1	Cecal microbiota only
2	Cecal microbiota plus *C. perfringens* 10^6^ cfu/mL plus phage #7 10^4^ pfu/mL
3	Cecal microbiota plus *C. perfringens* 10^6^ cfu/mL
4	Cecal microbiota plus phage #7 10^4^ pfu/mL
5	Cecal microbiota plus amoxicillin at 10 µg/mL
6	Cecal microbiota plus *C. perfringens* 10^6^ cfu/mL plus amoxicillin at 10 µg/mL
7	Cecal microbiota plus clindamycin at 10 µg/mL
8	Cecal microbiota plus *C. perfringens* 10^6^ cfu/mL plus clindamycin at 10 µg/mL

### gDNA extraction, library preparation, and sequencing

Genomic DNA was extracted from the samples using an Agowa/PurePrep protocol, as described in Wiese et al (Wiese et al., 2024). The library preparation for whole-genome sequencing was prepared using the Illumina DNA prep protocol according to the instructions of Illumina (Illumina DNA Prep Reference Guide, 1000000025416v10) as described in (Wiese et al., 2024). Raw sequence data, including metadata, were available through accession: PRJEB82952.

### Sequence data analysis

Kraken2 (Wood et al., 2019) (version 2.1.1) was used to taxonomically classify the metagenomic reads based on a reference database that contains RefSeq genomes of bacteria, fungi, archaea, and viruses (downloaded 24 February 2022) and phages. Kraken2 taxonomically classifies shotgun metagenomic reads using exact k-mer matches, providing high accuracy and speed. In short, the classifier matches every k-mer in the query (read) sequence to the lowest common ancestor (LCA) of all genomes in the database containing that k-mer. While Kraken2 classifies reads to the best-matching taxonomy, it does not estimate taxonomic abundances. Species- and genus-level relative abundance profiles were obtained using Bracken (Lu et al., 2017) (version 2.6.0), which is a highly accurate statistical method that uses Kraken2 output and provides accurate abundance estimates even when a sample contains two or more near-identical species.

Abundance bar graphs were created using R version 3.6.0 with ggplot2 version 3.1.1 (Wickham and Sievert, 2009). Shannon and Simpson values were calculated with the vegan package version 2.5-5 (Oksanen et al., 2019) using the diversity method. Chao values were calculated with the vegan package using the estimateR method. *p*-values were calculated with the ANOVA function of the carData package 3.0.2. Student’s *t*-tests with Benjamin–Hochberg correction were applied to compare groups. Principal coordinate analysis (PCoA) was performed using R version 3.6.0 with the vegdist function of the vegan package using the Bray–Curtis matrix. Results were visualized with ggplot2 version 3.1.1.

Differential abundance testing was performed with R version 3.5.1 and DESeq2 v 1.22.2 (Love et al., 2014). Selected samples were normalized together using the default normalization strategy of DESeq2. Significant results were selected based on a *p*-value (padj) <0.05. For visualization, significant results were additionally filtered on a minimum normalized baseMean of 1,000, and per condition, the average value was calculated. Heatmaps were created with the pheatmap package v1.0.10 with the option scale row on selected species with additional filtering of species with DESeq normalized baseMean above 1,000 and abundance in more than one sample. For all selected species of the differential expression, a boxplot of relative abundance was created using R version 3.6.0 with ggplot2 version 3.1.1.

### Quantitative PCR for quantification of *Clostridium perfringens* in i-screen

*Clostridium perfringens* present in the chicken i-screen was quantified using a specific *C. perfringens* qPCR. The DNA extracted used for metagenomic sequencing was used for this quantification PCR after 100 times of dilution. The primer/probe set used for amplification of a part of the 16S rRNA coding region of *C. perfringens* was 16S-Clperf-F 5′-GAACCTTACCTACACTTGAC-3′and 16S-Clperf-R 5′-CCACCTGTCACCTTGTCC-3′, and probe sequence 16S-Clperf was FAM-5′-TGCATTACTCTTAATCGAG-3′-MGB. The qPCR mixture was prepared, consisting of 12.5 µL of 2× Diagenode Master Mix, 1 µL of 10 µM 16S-Clperf-F, 1 µL of 10 µM 16S-Clperf-R, 1 µL of 5 µM 16S-Clperf, 4.5 µL of Milli-Q water, and 5 µL of DNA template derived from the i-screen sample. The qPCR was performed in the QuantStudio 5 Real-Time PCR System (Thermo Fisher Scientific, the Netherlands) with the following settings: 5 min preheating at 50°C, 10 min denaturation, and PCR initiation at 95°C followed by a two-step amplification with 40 cycles of alternating 15-s denaturation at 95°C and 60-s primer/probe annealing and complementary strand synthesis at 60°C. Serial dilution of isolated and quantified DNA from *C. perfringens* ATCC 13124 was used for trendline as a basis for quantification reference. Genome equivalents were calculated based on the genome size Cp = 3,256,683 bp 1 fg of gDNA isolated from *C. perfringens* equals 0.3 genome equivalents (Myers et al., 2006).

## Results

### Phage isolation and characterization

Ten dissected chicken intestines obtained from a slaughterhouse in Zevenhuizen and 12 surface water samples from the province of Utrecht, the Netherlands, were tested for the presence of *C. perfringens* phages. From these samples, only one sample taken from a pond in Amersfoort, overcrowded with ducks, yielded plaque-forming units on *C. perfringens* LMG 11264. Upon pure culturing of the phage, approximately 10^9^ plaque-forming units per mL (pfu/mL) were obtained. The other *C. perfringens* strain that showed plaque formation with the phages isolated from the pond with ducks were ATTC 13124, DSM 11781, and LMG 12225. No plaques were observed on LMG 12224 and LMG 10468 (see **Table 2**).

**Table 2:** Plaque formation by phage #7 on various *Clostridium perfringens* strains.

Bacterial species	Strain affiliation	Plaque formation	Remarks
*C. perfringens*	ATCC 13124 (TTC 05.0047)	**+**	Strain originally CN1491
*C. perfringens*	LMG 11264 (2011.022)	**+**	Cp strain originally CN1491
*C. perfringens*	DSM 11781 (2009.155)	**+**	Extremely small plaques
*C. perfringens*	LMG 12224 (2011.021)	**−**	
*C. perfringens*	LMG 12225 (2011.020)	**+**	
*C. perfringens*	LMG 10468 (2011.023	**−**	

Strain ATCC 13124 is an ancestor of strain LMG 112654 after custody from the NCTC collection and originating from the Wellcome Lab with strain number CN1491. The strain is also known as JCM 1290T, showing plaques with Cp bacteriophage CPQ1 (Mohammadi et al., 2022).

Seven randomly picked individual plaques cultured on *C. perfringens* LMG 11264 from the first cultivation round were subjected to genome sequencing and showed strong sequence similarity among the phages. This suggested that the phages were highly similar and perhaps originating from a single ancestor. The isolated phages were also highly similar to previously sequenced phages: OP753449.1, OP753450.1, OP753451.1, OP753452.1, and OP753453.1 (Wu, S. unpublished); MK017819.1 (Shin, D. and Ryu, S. Complete genome sequence of *Clostridium perfringens* phage CPD4, unpublished); KY206887.1 (Park, S.H., Paik, H.R., Jun, S.Y., Yoon, S.J., Kang, M.S., Kang, S.H. and Son, J.S. Virulent bacteriophage infecting *Clostridium perfringens* Clo-PEP-1, unpublished); MN417334.1 (Cho, J.-H., Kwon, J.-G., Kong, M., Ryu, S. and Lee, J.-H. Characterization and food application of a bacteriophage-derived endolysin and its cell-wall binding domain for biocontrol and rapid detection of *Clostridium perfringens* Clostridium phage CPAS-15, unpublished); and OP381444.1 (Tian, R. Isolation and identification of G-type *Clostridium perfringens* bacteriophages P21, unpublished).

### Microbiome composition overview—*in-vitro* study

**Figure 2:**
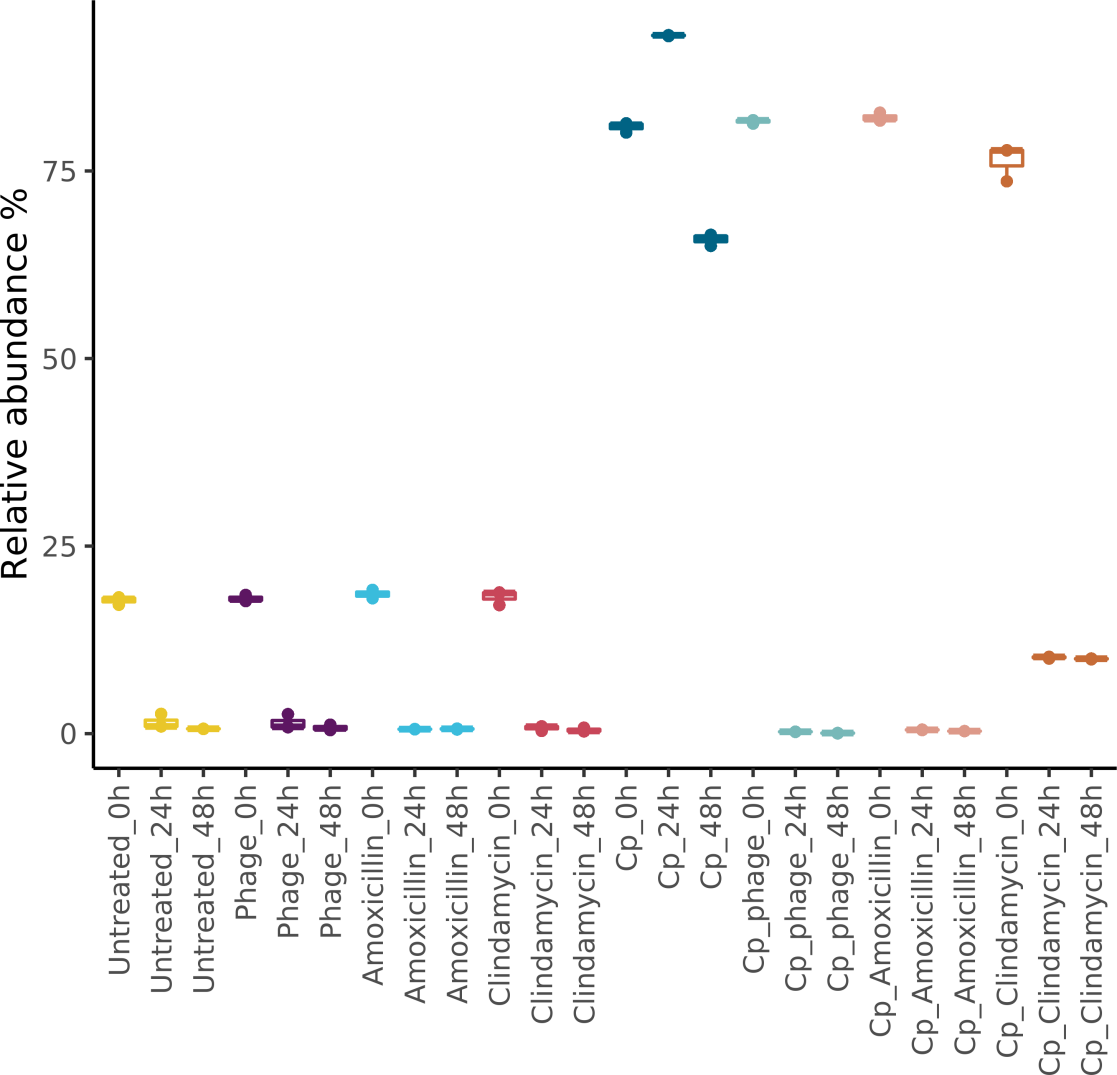
Relative abundance (%) of *C. perfringens* within the complex chicken cecal microbiota at the start of the experiment *T* = 0 and after 24 and 48 h of incubation. The different experimental culture conditions without *C. perfringens* spike (left side of the figure) (untreated) and with *C. perfringens* spike and antibiotics (clindamycin and amoxicillin) or *C. perfringens*-specific phage are indicated on the *x*-axis; conditions are color-coded.

In this study, we aimed to develop an *in-vitro* model of the chicken cecal microbiota with increased throughput to facilitate the testing of novel solutions against opportunistic pathogens, such as *C. perfringens*, next to established treatments with antibiotics. To achieve this, we collected and cultured a complex chicken cecal microbiota *in vitro* and spiked selected test conditions with a *C. perfringens* strain LMG 11264 (10^6^ cfu/mL) with and without concomitant treatment with a *C. perfringens*-specific phage #7, or the antibiotics clindamycin (10 µg/mL) or amoxicillin (10 µg/mL). To compare the levels of *C. perfringens* within the treated and untreated conditions, we displayed the relative abundance of *C. perfringens* across the experimental conditions and incubation time points (0, 24, and 48 h, designated as T0, T1, and T2, respectively) (**Figure 2**).

We have detected *C. perfringens* levels of an average of 18% at the start of the experiment in the control conditions without spiked *C. perfringens*, indicating the presence of an endgenous *C. perfringens* strain. Nevertheless, this endogenous strain did not proliferate *in vitro*, reflected by it's declining relative abundance throughout incubation time with 1% after 24 h and 0.7% after 48 hours of incubation. In conditions spiked with the *C. perfringens* LMG 11264 strain, the strains grew successfully within the complex microbiota throughout the incubation time and reached a relative abundance of 93% in the untreated conditions after 24 h, and the levels remained at approximately 66% after 48 h of incubation *in vitro* (**Figure 2**). We also determined the absolute abundance of *C. perfringens* within the chicken i-screen using the specific *C. perfringens* quantitative PCR. The *C. perfringens* LMG 11264 proliferated within the untreated complex microbiota and reached levels of approximately 10^8^ and 10^9^ genome equivalents per mL after 24 and 48 h of incubation, respectively. The addition of the *C. perfringens*-specific phage led to a decrease in the relative abundance of *C. perfringens* to approximately 0% within 24 h (T1), with levels remaining at 0% after 48 h of incubation. Amoxicillin had a similar inhibitory effect on *C. perfringens* growth, whereas the clindamycin treatment resulted in a less pronounced inhibition and reduced the growth of *C. perfringens* to a relative abundance of approximately 10% after 48 h of incubation (**Figure 2**).

**Figure 3:**
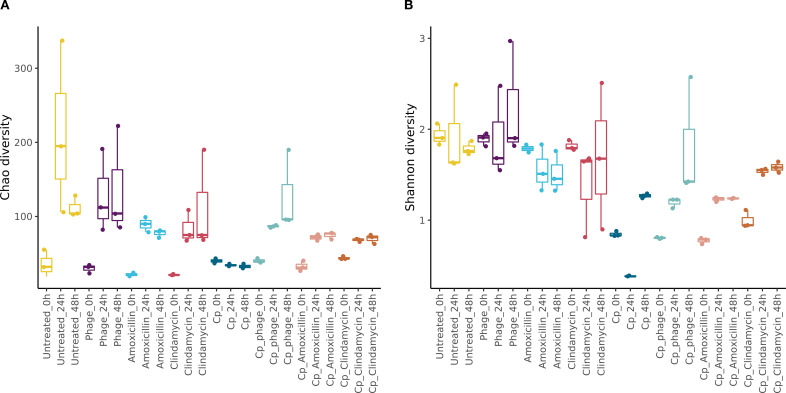
**(A)** Alpha diversity displayed as Chao diversity index as detected at *T* = 0, 24, and 48 h sampling time points within the i-screen conditions as displayed on the *x*-axis. **(B)** Alpha diversity displayed as the Shannon index as detected at *T* = 0, 24, and 48 h sampling time points within the i-screen conditions as displayed on the *x*-axis.

Furthermore, we also analyzed the alpha diversity within the different conditions (**Figures 3A, B**). We detected Chao indices of approximately 33.6 ± 10 at T = 0, and the spike with *C. perfringens* reduced the Chao index from 40 ± 3 at T = 0 to 33 ± 3 at T = 48 due to the overgrowth and dominance of *C. perfringens* within the complex microbiota.

The phage intervention alone did not impact the Chao index significantly when no *C. perfringens* (Cp) was spiked, indicating the phage did not proliferate without its specific host, whereas the antibiotics somewhat reduced the alpha diversity (after 48 h untreated: 111.67 ± 14.15, phage: 137 ± 74.22, amoxicillin: 77.33 ± 5.51, clindamycin: 83.67± 22.30 in the Chao index). When conditions were spiked with *C. perfringens*, after 48 h, 32.67± 3.06 taxonomic units were detected within those conditions, and while samples treated with antimicrobial solutions harbored a higher diversity after 48 hours (Cp and clindamycin: 70± 6.24, Cp and amoxicillin: 74.67 ± 4.93, Cp and Cp phage: 127 ± 54.56). In summary, for the Chao indices, we found a significant effect of amoxicillin (*p* = 0.040) or clindamycin (*p* = 0.000017) for the conditions without *C. perfringens* challenge when compared to the untreated control after 24 h; hence, at T1, this effect was not significant after 48 h. The phage treatment at 24 h did not differ significantly from the untreated condition at 24 h (*p* = 1). For the conditions spiked with *C. perfringens* (+Cp), significant differences were detected in the conditions with amoxicillin (+Cp) (*p* = 0.0057) or clindamycin (+Cp) (*p* = 0.0039) compared to untreated (+Cp) (at 24 h), and the effect was also significant with the phage within the *C. perfringens-*spiked condition (+Cp) treatment (*p* = 0.03). The following Shannon indices were detected for *C. perfringens* spiked samples 1.27± 0.03, for Cp amoxicillin treated 1.24 ± 0.01, and for Cp clindamycin treated conditions the Shannon index amounted to 1.58 ± 0.06 (**Figure 3B**).

### The intervention effects of pathogen, phage, or antibiotics on the microbial community composition throughout the incubation time

**Figure 4:**
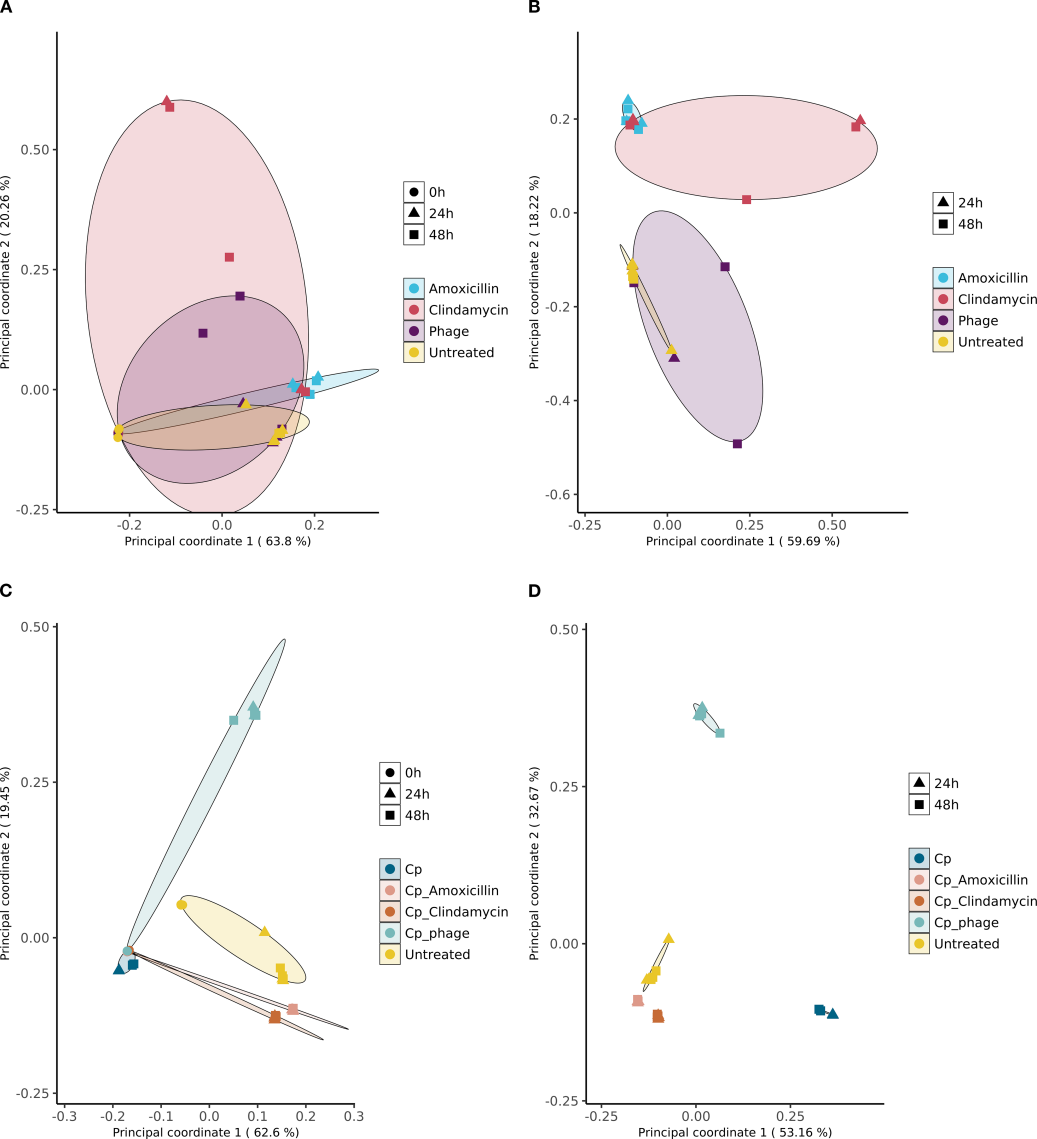
**(A–D)** The PCoA plots display an overview of microbiota samples (*n* = 3 per group). **(A)** For all time points and conditions and **(A, C)** for 24 and 48 h only. **(B, D)** Treatments spiked with *C. perfringens* (Cp) at *T* = 0 and after 24 and 48 h.

We created an overview of the beta diversity of the sample set within PCoA plots, displaying the diversity spread of the microbial communities within the test conditions without *C. perfringens* spike (**Figures 4A, B**) and with *C. perfringens* spike (**Figures 4C, D**), at the sampling time points *T* = 0, 24, and 48 h (**Figures 4A, C**) and sampling time points *T* = 24 and 48 h (**Figures 4B, D**). The analysis showed that treatments with the antibiotics clindamycin and amoxicillin (10 µg/mL) significantly changed the microbial community composition over time (**Figures 4A, C**), whereas the microbial communities treated with the phage did cluster close to the untreated conditions.

**Figure 5:**
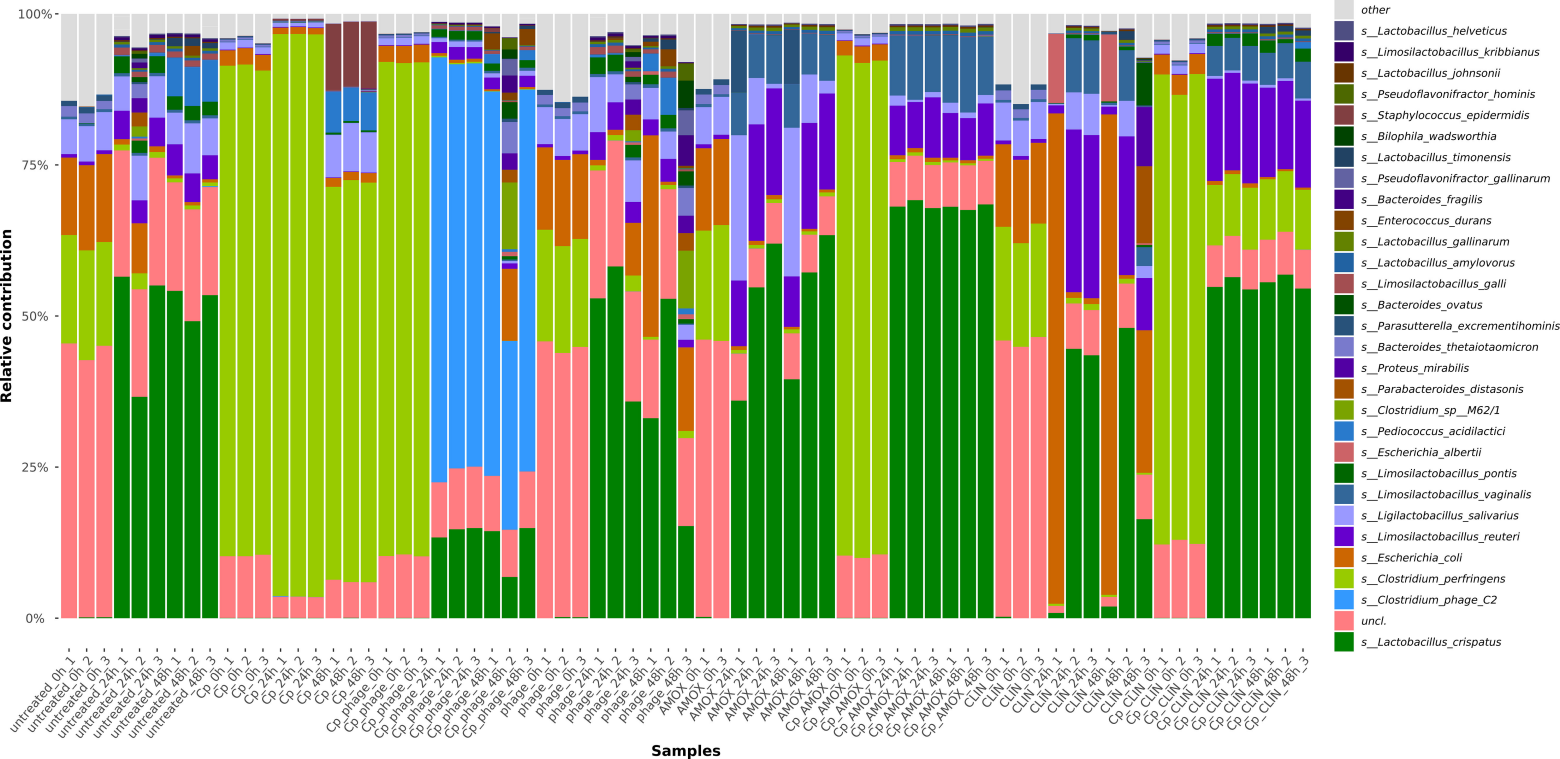
Relative abundances of the 30 most abundant species displayed in different colors; test conditions (*n* = 3) are indicated on the *x*-axis.

To investigate the specific changes in microbial taxa levels as induced by the different conditions, we visualized the relative abundances (%) of the 30 most abundant species within the samples at different sampling time points: *T* = 0, 24, and 48 h (**Figure 5**).

**Figure 6:**
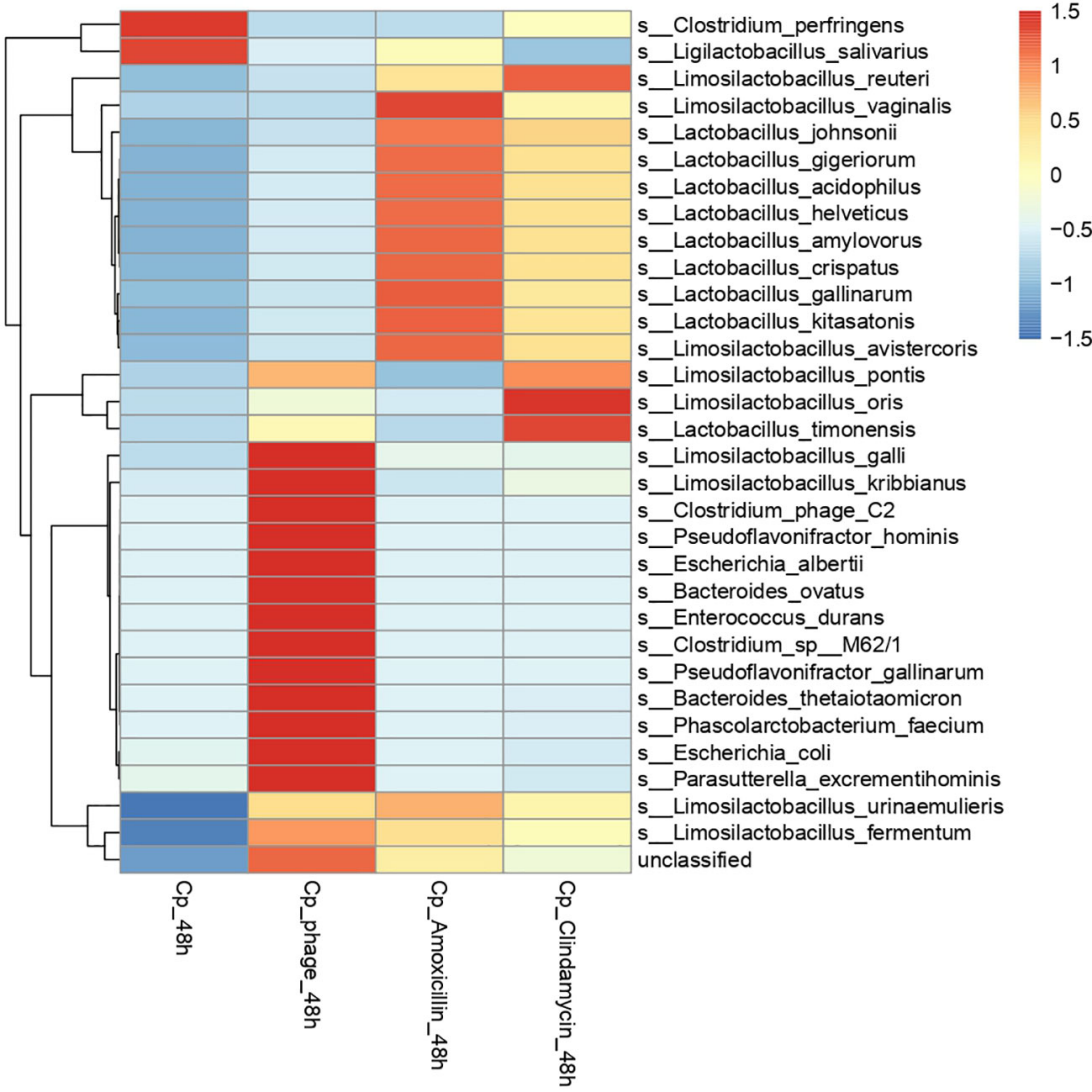
Heatmap showing the normalized log row-scaled average abundance of species-level taxa (padj < 0.05; baseMean > 1,000) and samples treated with phage, amoxicillin, or clindamycin at 48 h.

When left untreated, the Cp strain spiked into the culture condition was detected at high relative abundance 93% decreasing after 48 to 66%. The Cp phage reduced the Cp levels down to approximately zero per cen in relative abundance after 48 hours of incubation (**Figures 2**, **5**). Phage DNA was only detected at significant levels after 24 and 48 hours of incubation, when incubated with the host strain. The treatment with the antibiotic clindamycin also led to a significant reduction of spiked *C. perfringens* levels from 76% (0 h) to 10% (24 and 48 h), with a concomitant increase in the relative abundance of *Proteus mirabilis* and *Lactobacillus crispatus*. The amoxicillin treatment at 10 µg/mL inhibited the growth of *C. perfringens* after 24 and 48 h and led to a concomitant increase in *L. crispatus.* We performed a DESeq analysis to compare differential changes in microbial taxa related to the conditions spiked with *C. perfringens* after 48 h of incubation (CP_T2) and the conditions spiked and treated with phage or antibiotics after 48 h of incubation. **Figure 6** displays the significant fold changes found after 48 h of incubation compared to the untreated control.

When looking at the conditions with *C. perfringens* (Cp) spike alone without treatment, as depicted in the left-hand panel of **Figure 6** (Cp_48 h), there were significant positive fold changes in *C. perfringens* (indicated in red color) (**Figure 6**). Along with the significant fold change in *C. perfringens*, we detected a significant fold increase in *Ligilactobacillus salivarius* in this condition. When treated with the phage, these taxa decreased in abundance. There were also a minor negative fold changes in some of the lactobacilli and a positive fold increase in various taxa (indicated in red). A significant fold increase was, for instance, detected for *Escherichia* species.

The antibiotic treatment with amoxicillin reduced the growth of *C. perfringens* and some other taxa, e.g., *Escherichia* species. It facilitated a concomitant increase in numerous taxa, such as some *Lactobacillus* spp. and some *Limosillactobacillus* spp. Similar effects were detected for the clindamycin-treated condition.

## Discussion

Chickens provide eggs and meat and play a pivotal role in human life (Wang et al., 2024). Pathogens such as *C. perfringens* may impact chicken health and productivity, and the rise of antibiotic resistance has led to the increased necessity for the development of alternative solutions against enteropathogenesis and sustainable health and production outcomes (M’Sadeq et al., 2015). The commensal microbiota that populates the chicken gastrointestinal tract exerts various beneficial roles for the host’s health. It impacts growth, development, and health via effective feed conversion and essential metabolite production, such as short-chain fatty acids (Deryabin et al., 2024). The endogenous microbiota is also a key player in the colonization resistance against pathogens (Card et al., 2017; Rychlik, 2020). Therefore, it is desirable to support a beneficial commensal intestinal microbiome for optimal health when developing novel applications against enteric pathogens. Despite the existence of *in-vivo* models for the study of colonization, infection by *C. perfringens*, and the *C. perfringens*-associated necrotic enteritis in poultry (Myers et al., 2006; Deryabin et al., 2024; Wang et al., 2024), *in-vitro* models allow us to examine the effects of interventions on the microbiota under controlled experimental conditions without as many of the ethical questions associated with *in-vivo* studies (Feye et al., 2020; Rychlik, 2020).

*In-vitro* gut models facilitate the study of intervention effects on pathogen outgrowth and overall gut microbial community dynamics, allowing the evaluation of the treatment specificity and efficacy, as well as the evaluation of more general effects on the microbial community composition and function (Wiese et al., 2024). In this study, we presented the chicken i-screen experimental setup, which allows us to test different solutions against enteropathogens such as the *C. perfringens* strain LMG 11264 within a complex chicken microbiota *in vitro*. When left untreated, the *C. perfringens* strain proliferated well within the complex microbiota throughout the incubation time, reaching levels of approximately 10^9^ genome equivalents per g intestinal content (results not shown). Similar levels have been described in animals *in vivo* with reported necrotic enteritis and enumerations of cell-forming units of 10^6^–10^8^ cfu/g (Timbermont et al., 2010; Timbermont et al., 2011; Mora et al., 2020).

In this study, we successfully simulated the pathogen challenge with the *C. perfringens* strain LMG 11264 within the chicken i-screen *in vitro.* Due to the 96-well plate-based experimental approach and relatively high experimental throughput, it was possible to test the specificity and efficacy of different solutions against toxigenic *C. perfringens* in replicates and in parallel throughout the incubation time. The increased throughput of the i-screen experimental setup can also facilitate the testing of different dosages/or phage titer effects and/or combinations of therapies of antibiotics and phages or phage cocktails. Furthermore, several complex microbiotas can be included in such studies to expand insights into the specificity and efficacy of the therapies. Additionally, some of the mechanistic aspects of phage treatments, such as the kinetics of self‐replicating pharmaceuticals, as summed up by Payne and Jansen (Payne and Jansen, 2000), still need further exploration, and *in-vitro* models provide ample possibilities to study the host–phage dynamics with a focus on inoculation levels and host–phage exposure time points. *In-vitro* models are recognized as powerful tools to unravel the effects of enteric pathogens on the gut microbiota (Calvigioni et al., 2023), and our 96-well plate-based screening demonstrates an efficient and flexible experimental approach.

The screening provides cost-effective insights, often hampered in more complex *in-vitro* models operating with a larger working volume and lower throughput. In this study, the background of endogenously present *C. perfringens* decreased throughout the incubation time to approximately 0%. The endogenous *C. perfringens* did not grow out throughout the incubation time, perhaps due to its presence as spores that did not germinate under the implemented culture conditions. In the case of the presence and persistence of multiple *C. perfringens* strains, additional qPCR assays and bioinformatic analyses can be included in the study design for the differentiation of strains and evaluation of intervention specificity.

Amoxicillin is one of the penicillin derivatives effective against susceptible strains of various Gram-positive and Gram-negative bacteria. Amoxicillin is administered when necrotic enteritis is suspected in poultry, and it is the drug of choice for preliminary treatment until confirmation. Furthermore, penicillin derivatives such as amoxicillin are also implemented in the treatment of *Escherichia coli* and *Salmonella* infections, as well as chronic respiratory disease in poultry (Khatun et al., 2020; Naeem et al., 2024).

The *C. perfringens* strain implemented in this study was sensitive to amoxicillin, and its growth was inhibited at 10 µg/mL of amoxicillin and, to a lesser extent, also clindamycin at the same concentration. The selected phage #7 was effective against the spiked *C. perfringens* strain *in vitro* and reduced *C. perfringens* strain levels with similar efficacy as 10 (µg/mL) of amoxicillin. The supplementation of the media with the antibiotics amoxicillin or clindamycin exerted changes in the gut microbial community composition. The phage treatment did not shift the gut microbial community composition when supplied without its host *C. perfringens* strain. Host factors that govern bacteriophage infectivity and specificity have been identified genome-wide (Chitboonthavisuk et al., 2024). A comparative genome analysis between the *C. perfringens* strain LMG 11264 versus the endogenous *C. perfringens* could be conducted for additional information on phage insensitivity, but this was beyond the scope of this study. In addition to the gut microbial community dynamics detected in this study, *in-vitro* studies may also facilitate insights into microbial metabolite levels, such as SCFA, which are relevant for colonization resistance and health (Wiese et al., 2024).

Furthermore, culture-independent metagenomic approaches are enhancing our understanding of the chicken gut microbiota and its functional gene repertoire, as well as the antimicrobial resistance genes present in the chicken gut (Feng et al., 2021). Feng et al. constructed a gene catalog by integrating public chicken gut microbiome samples from 10 countries. They found *Lactobacillus aviarius* and *L. crispatus* to be the most common lactic acid bacteria in the chicken gut (Feng et al., 2021), and resistance genes were present in different microbial taxa. In line with those insights, we also detected *L. crispatus* in our study, which significantly increased in relative abundance after the antibiotic treatment with amoxicillin. Many *Lactobacillus* strains and species may carry antibiotic resistance genes that may be transferable to human and animal pathogens (Kaszab et al., 2023). In this context, the study of Dec et al. is relevant; they reported on the susceptibility to antibiotics and the presence of drug resistance genes in 88 *Lactobacillus* isolates derived from chickens (Dec et al., 2017). Metagenomic insights into chicken gut antibiotic resistomes and microbiomes (Yang et al., 2022) emphasize the need for alternative therapies. In our study, we also found an indication of potential antibiotic resistance within *Lactobacillus* species, which increased significantly in relative abundance within antibiotic-treated conditions (**Figure 6**).

*In vitro*, tools can also be used to study the transfer of AMR gene-harboring plasmids (Card et al., 2017; Anjum et al., 2018), emphasizing the necessity to further develop and apply *in-vitro* experimental approaches to understand animal health and processes in the animal gut microbiota. In this context, screening approaches with higher throughput are relevant as they facilitate testing various interventions and microbiotas in parallel, e.g., chicken microbiotas from different farms for ecologically relevant results.

The gut microbiota is a diverse multi-kingdom ecosystem constituted by bacteria, archaea, and viruses, and metagenomic analyses of the chicken multi-kingdom microbiome, including bacterial, archaeal, and viral genomes, are just emerging and paving the way for more advanced and comprehensive insights into the actual gut health dynamics relevant for poultry. For instance, Wang et al. found diverse auxiliary metabolic genes and antibiotic resistance genes to be carried by viruses (Wang et al., 2024). They constructed an up-to-date and most extensive chicken gut-derived gene catalog based on integrated metagenome assembled genomes (MAGs) and viral genomes (Wang et al., 2024). Such metagenomic analyses have immense potential in combination with *in-vitro* gut model systems, facilitating in-depth functional insights that may pave the way for microbial interventions for better chicken gut and overall health.

Based on the discussion, representative models and the use of phages or their endolysins support the development of new strategies against enteropathogens like *C. perfringens*, taking also the additive and synergistic effects of different measures into account.

## Data Availability

The data presented in the study are deposited in the ENA repository, accession number PRJEB82952.
